# Blue-Light-Blocking Lenses Ameliorate Structural Alterations in the Rodent Hippocampus

**DOI:** 10.3390/ijerph191912922

**Published:** 2022-10-09

**Authors:** Elizebeth O. Akansha, Bang V. Bui, Shonraj B. Ganeshrao, Pugazhandhi Bakthavatchalam, Sivakumar Gopalakrishnan, Susmitha Mattam, Radhika R. Poojary, Judith S. Jathanna, Judy Jose, Nagarajan N. Theruveethi

**Affiliations:** 1 Department of Optometry, Manipal College of Health Professions, Manipal Academy of Higher Education, Manipal 576104, India; 2 Department of Optometry & Vision Sciences, School of Health Sciences, University of Melbourne, Parkville, VIC 3010, Australia; 3 INSOFE Education, upGrad-INSOFE, Hyderabad 500034, India; 4 Department of Anatomy, Melaka Manipal Medical College (Manipal Campus), Manipal Academy of Higher Education, Manipal 576104, India; 5 Department of Physiology, Kasturba Medical College, Manipal Academy of Higher Education, Manipal 576104, India

**Keywords:** light-emitting diodes (LEDs), hippocampus, behavior analysis, retinal damage, blue-light blocking lenses (BBLs)

## Abstract

Evidence suggests that prolonged blue-light exposure can impact vision; however, less is known about its impact on non-visual higher-order functions in the brain, such as learning and memory. Blue-light-blocking lenses (BBLs) claim to reduce these potential impacts. Hence, we assessed structural and functional hippocampal alterations following blue-light exposure and the protective efficacy of BBLs. Male Wistar rats were divided into (n = 6 in each group) normal control (NC), blue-light exposure (LE), and blue-light with BBLs (Crizal Prevencia, CP and DuraVision Blue, DB) groups. After 28 days of light exposure (12:12 light: dark cycle), rats were trained for the Morris water maze memory retention test, and brain tissues were sectioned for hippocampal neuronal analysis using Golgi and Cresyl violet stains. The memory retention test was significantly delayed (*p* < 0.05) in LE compared with DB groups on day 1 of training. Comparison of Golgi-stained neurons showed significant structural alterations, particularly in the basal dendrites of hippocampal neurons in the LE group, with BBLs significantly mitigating these structural changes (*p* < 0.05). Comparison of Cresyl-violet-stained neurons revealed significantly (*p* < 0.001) increased degenerated hippocampal neurons in LE rats, with fewer degenerated neurons in the CP lens group for CA1 neurons (*p* < 0.05), and for both CP and DB groups (*p* < 0.05) for CA3 neurons. Thus, in addition to documented effects on visual centers, high-level blue-light exposure also results in degeneration in hippocampal neurons with associated behavioral deficits. These changes can be partially ameliorated with blue-light-blocking lenses.

## 1. Introduction

Many biological functions require a light–dark cycle to synchronize circadian rhythms [1,2]. The amount and spectral composition of light that predominates in our environment can impact circadian rhythms. Those wavelengths that impact circadian rhythms the most are those in the shorter wavelength band, or blue-light [3,4]. Light sources that contain a higher proportion of short wavelengths are increasingly prevalent in our visual world. Light-emitting diodes (LEDs) are becoming more widespread [5] due to their long-life and higher efficiency [6]. LEDs can emit a broad range of wavelengths [6], containing a high peak at short wavelengths between 400 and 495 nm [7]. The wavelengths that carry the highest energy and are potentially most hazardous to the retina lie between 415 and 455 nm [8,9]. Excessive use of LED light and back-lit electronic screens might impact both visual and non-visual functions [10].

Excessive exposure to very high levels of short-wavelength light can lead to phototoxicity (“blue-light” damage) [11], which is categorized as a Class I or II type injury. Class I damage (photoreceptor damage) occurs after exposure to low illumination levels for days to weeks [12], whereas class II damage (retinal pigmented epithelium (RPE) damage) occurs after exposure to high levels of illumination for minutes to hours [13]. Light damage results from the excessive production of toxic reactive oxygen species leading to photo-chemical injury and the apoptosis of photoreceptors, RPE cells, and inner retinal neurons such as retinal ganglion cells [8,13,14,15,16]. Light damage can also affect intrinsically photosensitive retinal ganglions cells (ipRGCs), which play a pivotal role in photoentrainment by transmitting the light signals to the suprachiasmatic nucleus (SCN) through the retinohypothalamic tract [17,18,19,20]. 

ipRGCs also innervate the ventromedial prefrontal cortex (vmPFC) and transmit light signals to the perihabenular nucleus (PHb) for mood regulation [21]. The hippocampus is closely associated with the prefrontal cortex (PFC), playing a key role in learning and memory [22]. More specifically, Cornu Ammonis neuron-1 (CA1) and CA3 of the tri-synaptic circuit of the hippocampus are responsible for temporal processing and memory storage [23]. As such, changes in learning, memory, cognition, and mood induced by excessive blue-light exposure during the light–dark cycle [24] may involve alterations to neurons in the hippocampus [25]. 

The potential for excessive light exposure to disrupt circadian rhythms as well as visual and non-visual function [1,2,26,27,28] has led eye care professionals to consider whether the attenuation of short-wavelength light exposure may be of clinical benefit [29]. Commercially available BBLs, in the form of spectacle lenses or intraocular lenses (IOLs), have been claimed to reduce eyestrain, enhance sleep quality, prevent retinal toxicity, and slow the progression of age-related macular degeneration (AMD) [30]. In a previous study using a rodent model, BBLs provided protection for visual cortex pyramidal neurons against chronic exposure to high levels of short-wavelength light from LEDs [31]. However, to the best of our knowledge, the impact of short-wavelength light exposure on animal behavior and hippocampal neuron morphology is yet to be examined. This study explored the effects of high-level blue-light exposure on hippocampus neurons and animal behavior using a spatial learning task and considered whether BBLs modify the effects of this blue-light exposure. 

## 2. Methodology

### 2.1. Ethical Statement

This study was approved by the “Institutional Animal Ethics Committee (IAEC/KMC/36/2020)” of the Manipal Academy of Higher Education, Manipal (MAHE, Manipal, India) and was carried out in accordance with CPCSEA guidelines (CPCSEA No: 94/PO/Re Bi/5/99/CPCSEA). Following approval, healthy adult male Wistar rats (n = 24, 8 weeks old) were procured from the Central Animal Research facility (MAHE).

### 2.2. Standardization of Light Exposure and Laboratory Setup

Animals were housed in a controlled laboratory (Central Animal Research facility, MAHE) in sterile polypropylene cages with paddy husk bedding, with water and a standard pellet diet available ad libitum [32]. Animals were divided into four groups (n = 6 in each group): (1) normal controls (NC), (2) blue-light exposure (LE), (3) blue-light exposure with blue-light-blocking lens I—Duravision Blue (DB, Carl Zeiss, Oberkochen, Germany), and (4) blue-light exposure with blue-light-blocking lens II—Crizal Prevencia (CP, Essilor, Charenton-le-Pont, France). Animals in the NC group were maintained under a normal laboratory environment (450–500 lux; 12:12 h light/dark cycle on at 8 am), whereas the animals in the LE group were exposed to short-wavelength blue-light from LED for 12 h a day for 28 days (12:12 h light/dark cycle on at 8 am). As previously described, the blue LEDs (Ack LED panels, Epistar, 3 W, ES-EMBCF30C, F Series, InGan series, blue LED chip, Epistar corporation, Hsinchu, Taiwan) were fixed to the cage top, which sat at a height of 52 cm above the cage (L = 108 cm, W = 78 cm) and provided illumination of 450–500 lux (at the cage bottom), as measured with a spectrometer (Asensetek Lighting Passport Pro, New Taipei City, Taiwan). Light levels were averaged from measurements of both horizontal and vertical planes [33]. Retinal irradiance was calculated as previously described [34]. For the lens treatment groups, the BBLs (CP and DB) were fixed and sealed to completely cover the LEDs.

### 2.3. Behavioral Assessment Post-Light Exposure

The Morris water maze (MWM) test is a well-accepted hippocampus-dependent task and the most widely used paradigm for assessing spatial learning and memory [35]. The water maze apparatus consists of a circular pool (1.5 m diameter) divided into four imaginary quadrants. The pool is filled with water at a temperature of 18–23 °C to a depth of 40 cm. A 4″ × 4″ platform is immersed 1 cm below the water surface in one of four randomly selected quadrants (“target quadrant”) called the island zone. The platform is hidden by adding non-toxic white tempura paint to make the water opaque [32,35,36]. If animals do not discover the hidden platform using visual cues within 120 s, they were guided to the platform and left there for 20 s [36]. 

A visible cue (a symbol ‘+’; 10 cm H = 10 cm, W = 10 cm, 100% contrast black on white paper) was placed in one of the quadrants to facilitate spatial orientation for the animal [35]. A video camera (640 × 480 pixels Logitech B525 HD Webcam, Lausanne, Switzerland) was placed above the center of the pool to record the rat swimming. Computer tracking software (ANY-maze version 4.82, Stoelting Co. Wood Dale, IL, USA) pinpointed the rat’s center of gravity at 10 Hz. 

At 28 days after light exposure, rats underwent training for four consecutive days with four trials per day. Rats were habituated for 5–10 min in the testing room prior to testing [32,35]. During each trial, rats were placed facing a side wall of the pool at randomly selected starting positions. The time taken to reach the hidden platform was recorded [36]. On the fifth day, animals were subjected to one session of a 60 s memory retention test, where the hidden platform was removed. The total time taken to reach the target quadrant during the four consecutive training days was recorded. Data were compared across all groups. 

### 2.4. Structural Assessment

#### 2.4.1. Dendritic Arborization Assessment Using Golgi Stain

Two days after the final behavioral assessment, animals were sacrificed with an overdose of Pentobarbitonol (intraperitoneal, 100 mg/kg, Virbac AH, Inc., Westlake, TX, USA) and xylazine (Proxylaz^®^ 10 mg/kg, Prodivet pharmaceuticals, Raeren, Belgium). The brain was dissected into right and left hemispheres, and one hemisphere was impregnated in freshly prepared Golgi–Cox fixative for 21 days (the stain was changed every 5 days). Golgi–Cox fixative was prepared with mercury chloride (Medilise Chemicals, KRL/KNR/00087/2003, Azhikode Kannur, Kerala, India), potassium chromate (Spectrum Reagents and Chemicals Pvt. Ltd., Edayar, Kerala, India), and potassium dichromate (Spectrum Reagents and Chemicals Pvt. Ltd., Edayar, Cochin, Kerala, India), mixed using a magnetic stirrer (ROTEK magnetic stirrer, Ernakulam, Kerala, India). 

Hippocampal sections 150 µm in thickness were obtained using a sledge microtome (Radical scientific equipment, Pvt. Ltd., Ambala Cannt, Haryana, India). Sections were stored in tissue cassettes (Leica-LP-C-Biopsy Cassettes, Wetzlar, Germany) and further treated with 5% sodium carbonate solution for 20 min to make neurons visible. Tissue sections underwent dehydration in ascending orders of ethyl alcohol (Ethanol, UN No.: 1170), starting with 70% for 10 min (2 washes, 90% for 10 min (2 washes), and then pure alcohol (99.9%) for 10 min (3 washes). Finally, sections were cleared with sulphur-free xylene (Spectrum, New Brunswick, NJ, USA) to make the tissues transparent. Sections were then mounted on dry microscopic slides (BIOCRAFT, 26 × 76 MM, CAT NO. 7101, New Agra, Agra, India) using Dibutylphthalate Polystyrene Xylene (Merck, DPX; Sigma-Aldrich, St. Louis, MI, USA). As previously described [37], Motic Images Plus 2.0 ML software (Motic Red 200 microscope, Motic, Kowloon, Hong Kong) was used to capture images (30 per neuron) of stained neurons in steps of 5 µm using a light microscope with a digital microscope camera (Moticam 580, 5.0 MP; Model no. 12000425, Motic). From each tissue sample, 12 hippocampal neurons (CA1 and CA3) were manually traced by a grader who was blind to the study group. During the tracing of neurons, the completeness of staining of an individual neuron, background staining, uniformity of neuronal staining, clarity in staining of dendritic spines, and any artefacts were taken into consideration. Dendritic arborization, which included apical and basal branching points and intersections, was quantified individually using Sholl’s concentric circle method [38] by two of the investigators, and the mean value was recorded. Apical and basal dendrites have different electrical conductivity values, placements, sizes, and neurotrophic responses [39,40,41]. Apical dendrites run distally from the soma, whereas basal dendrites run to a shorter distance from the soma. However, the extent of their different functions requires further investigation [42].

#### 2.4.2. Histopathological Assessment Using Cresyl Violet Stain

The other hemisphere of the brain was harvested and preserved in 10% formalin for 6 days. Subsequently, it was processed in ascending grades of ethyl alcohol (99.9% Ethanol, UN No.: 1170) in the order of 50% and 70% for 24 h each; 90% and 100% for 12 h each; and two parts of xylene for 30 min each. Coronal sections were cut and placed in embedding rings with paraffin wax (Spectrum, Block form, Congealing point 60–62 °C) using an embedder machine (LEICA EG1150C). Tissues were frozen at a temperature of −20 °C for 48 h and then placed in a rotary microtome (LEICA RM2245) for trimming (OptiCut™ LPX Blades, low profile, ultra-thin, 0.010′ blade thickness, Polytetrafluoroethylene-coated, StatLab, McKinney, TX, USA). After trimming, 10 µm sections were taken and placed in a water bath (LEICA HI 1210), which was maintained at a temperature of 60 °C. The sections were removed using microscopic slides (Biocraft Scientific Systems, 26 × 76 mm, CAT NO. 7101, New Agra, Agra, India) with egg albumin applied on the surface from the water bath using forceps and placed on a hot plate (LEICA HI 1220) until the wax was melted. Sections were stained with Cresyl violet and prepared by dissolving 100 mg (Sigma-Aldrich chemicals, Merck, St. Louis, MI, USA) in distilled water (100 mL) with 10% glacial acetic acid (0.5 mL). The pH of the stain was measured (pH 510, pH/mV/°C meter, Eutech Instruments Pty Ltd., Singapore) and maintained at 3.4. Before use, the stain was filtered through Whatman filter paper (125 mm Ø, Cat No 1001 125 Whatman™, Little Chalfont, Buckinghamshire, UK). Sections were then deparaffinized in xylene for 15 min, then hydrated through a descending series of alcohol washes (99.9% Ethanol, UN No.: 1170) from 100%, 90%, 70%, to 50%, each lasting 2 min, with a final distilled water wash of 5 min. Sections were then processed in pre-warmed 0.1% Cresyl violet stain for 30 min at 60 °C and cooled to room temperature. They were further placed in distilled water for 5 min and dehydrated using an ascending series of alcohol washes, from 70%, 80%, 90%, to 100%, each lasting 2 min. Before mounting with DPX (Merck, Sigma-Aldrich, St. Louis, MI, USA), sections were cleared with xylene for 2 min. Images were captured using (Motic Images Plus 2.0 ML) [37] under 20× magnification using a light microscope with a digital microscope camera (Moticam 580, Motic). For each section, 100 neurons in CA1 and CA3 hippocampal regions were counted and examined for any morphological changes by two investigators who were blind to the study group, and the mean value was recorded. 

#### 2.4.3. Statistical Analysis

Functional and structural parameters for Golgi-stained neurons were compared across groups using R (version 3.6.3, Massachusetts Institute of Technology (MIT), Cambridge, MA, USA). Analysis of histopathological parameters derived from Cresyl violet staining was performed using IBM Statistical Packages for the Social Sciences (SPSS version 21.0, IBM, Chicago, IL, USA). Comparisons across groups and times for MWM parameters and structural changes with Golgi stain in the CA1 and CA3 neurons were performed using two-way analysis of variance (ANOVA), because there were two independent variables with multiple levels to compare the means across the groups for the impact of light exposure, where the data are represented as the mean ± SD [43]. Post hoc analysis was performed to report significance between the groups if any were found. Comparisons across groups for Cresyl-violet-stained neurons were performed using Kruskal–Wallis (non-parametric equivalent of one-way ANOVA) test because there was one independent variable with two levels [44]. 

## 3. Results 

### 3.1. Behavioral Analysis

**Figure 1:**
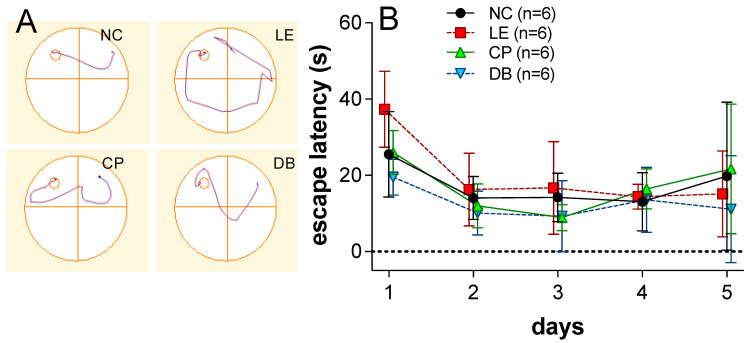
(**A**) Representative track plots for an animal from the four groups. NC, left top corner; LE, right top corner; CP, left bottom corner; and DB, right bottom corner (right side). (**B**) Group (±SD) escape latency (s) on the 5 consecutive trial two-way ANOVA indicated a significance difference (*p* < 0.05) on day 1 to day 3, but not days 4 or 5.

Example swim tracks show the search path taken by an animal to reach the hidden platform (Figure 1A), with shorter pathways representing better spatial learning and memory. The mean escape latencies (in seconds) to reach the hidden platform was compared across groups for all trial days and the final memory retention test. A statistically significant difference was found using two-way ANOVA when comparing across sessions (F_4, 392_ < 0.0001). Overall, the LE group without BBLs had longer latencies across days 1 to 3. At day 1, the two BBL groups were similar to the control group, with Tukey’s post hoc analysis revealing that the DB lens group was better than the LE group (*p* = 0.024). No post hoc differences were found at the other time points. Indeed, at Day 4, all groups returned similar escape latencies (Figure 1B). Regarding memory retention, there were no significant post hoc differences between groups.

### 3.2. Structural Analysis

#### 3.2.1. Morphological Assessment Using Golgi Stain

**Figure 2:**
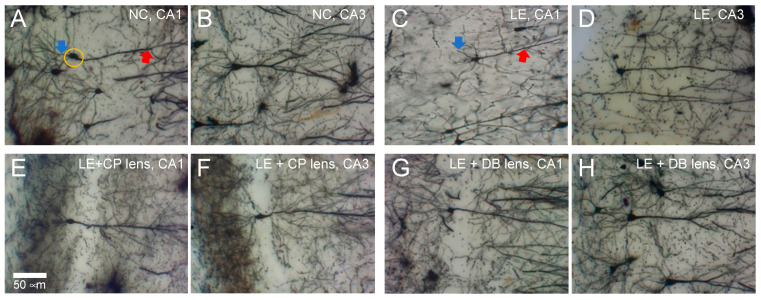
Representative micrographs of Golgi–Cox-stained CA1 and CA3 neurons. (**A**) Normal Control, NC (CA1) apical (red arrow) and basal dendrites (blue arrow) are indicated along with the soma (orange circle). (**B**) NC (CA3). (**C**) Light exposure, LE (CA1). (**D**) LE (CA3). (**E**) Light exposure with the CP lens (CA1). (**F**) LE + CP (CA3). (**G**) Light exposure with the DB lens (CA1). (**H**) LE + DB (CA3).

**Figure 3:**
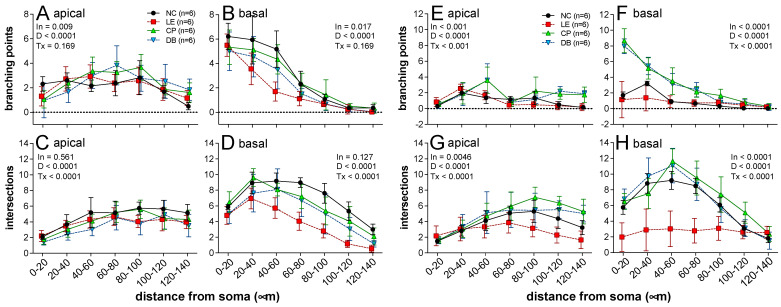
Averaged (±SD) apical and basal branching and intersection points for CA1 and CA3 neurons across all the groups for every 20 microns ranging from 20 to 140 µm. Values from total of 12 hippocampal neurons (CA1 and CA3; in each region, there 2 neurons from n = 6 animals/group). Significance determined using Tukey’s post hoc analysis is denoted by “ln”, which indicates interactions between treatment group (Tx) and distance (D).

Figure 2 shows representative photomicrographs of Golgi-stained neurons in areas CA1 and CA3 of the hippocampus. These cells were manually traced by a grader who was blind to the study group, which enabled the number of apical and basal branching points and intersections to be quantified and compared between groups. Group data are summarized in Figure 3.

Data across all groups for the number of apical and basal dendrites as functions of distance from the soma were compared using two-way ANOVA. For CA1 neurons (Figure 3A–D), significant interactions between treatment and distance were found for basal branching points (Figure 3B), as well as the number of apical (Figure 3C) intersections. In general, there were fewer basal branch points and intersection points in the light exposure group compared with controls. These changes were partially ameliorated by the BBLs. Specifically, for CA1 neurons, significant differences were found between control, exposure, and treatment groups in dendritic arborization parameters: apical branching points (Figure 3A, F18, 140 = 0.009, *p* < 0.05) and basal branching points (Figure 3B, F18, 140 = 0.017, *p* < 0.05) (Figure 2). 

A similar pattern was seen for CA3 neurons (Figure 3E–H). Significant interactions were noted for all parameters. In particular, light exposure resulted in fewer intersection points in both apical (Figure 3F) and basal (Figure 3G) dendrites, as well as fewer apical (Figure 3E) and basal branching points (Figure 3F). No deficit in intersection points (Figure 3G,H) was seen with the BBLs in place.

#### 3.2.2. Histopathological Analysis Using Cresyl Violet Stain

**Figure 4:**
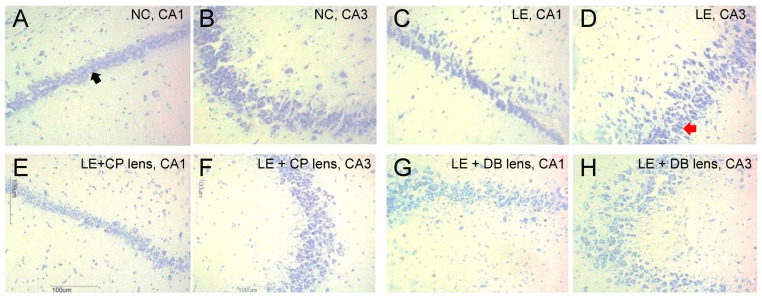
Representative micrograph of Cresyl-violet-stained CA1 and CA3 neurons. (**A**) Normal control CA1 region. Black arrow indicates a healthy neuron. (**B**) Normal control CA3 region. (**C**) light exposure (LE) CA1 region. (**D**) LE CA3 region. Red arrow indicates a degenerated neuron. (**E**) CP lens CA1 region. (**F**) CP lens CA3 region. (**G**) DB lens CA1 region. (**H**) DB lens CA3 region.

Healthy and degenerated CA1 and CA3 neurons were differentiated based on their structure. Figure 4 shows a representative photomicrograph of Cresyl-violet-stained CA1 and CA3 neurons across the various groups. Healthy neurons had a clearly defined cell body with dense nuclei and pale surrounding cytoplasm (Supplementary Figure S1A), whereas degenerated neurons had a dark pyknotic nucleus and irregular cell bodies (Supplementary Figure S1B). A Kolmogorov–Smirnov test revealed that neuron counts were not normally distributed (*p* < 0.001). Hence, a non-parametric Kruskal–Wallis test was performed to compare between groups, with chi-squared post hoc comparisons. Light exposure caused a reduction in healthy neurons and an increase in unhealthy neurons (Figure 4C,D). The addition of BBLs resulted in a pattern of healthy and unhealthy neurons that was similar to the normal control group (Figure 4E–H).

**Figure 5:**
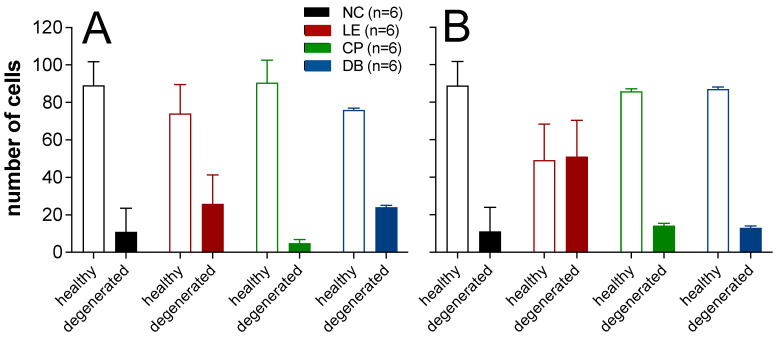
(**A**) Comparison of the healthy and degenerated CA1 and (**B**) CA3 neurons. Data for n = 6 animals from each group are shown as the mean ± SD for healthy and degenerated neurons.

As summarized in Figure 5, the CA1 region showed statistically significant differences (H = 39.15, *p* < 0.001) for healthy and degenerated neurons between groups (Figure 5A). Post hoc test revealed the same significance between LE and CP groups (i-j = −21.00, *p* = 0.003) and between CP and DB groups (i-j = 19.11, *p* = 0.009). The CA3 region showed a similar trend, where there was a significant difference (H = 38.98, *p* < 0.001) (Figure 5B) between healthy and degenerated neurons. Post hoc tests revealed significant differences between LE and NC (i-j = 39.89, *p* < 0.001), LE and CP (i-j = 36.88, *p* < 0.001), and LE and DB (i-j = 38.00, *p* < 0.001) groups. Overall, the number of healthy neurons was reduced, and the number of degenerated neurons increased in the LE group in comparison with NC, CP, and DB groups for both CA1 and CA3 neurons (Figure 5). 

## 4. Discussion

This study found that 28 days following exposure to high levels of short-wavelength light, there was a deficit in spatial learning on day 1, which was ameliorated with blue-light-blocking lenses. This functional deficit was associated with changes to the complexity of hippocampal neurons, particularly the basal dendrites in neurons in CA1 and CA3 regions. There was also evidence of more cells with pyknotic nuclei, particularly in CA3. Consistent with better function (at day 1) with BBLs, the lenses ameliorated some of these structure deficits induced by exposure to high levels of short-wavelength light.

In a previous study employing the same light exposure protocol, the retina was thinned and neurons in layer 5 of the visual cortex were negatively impacted, particularly with reductions in basal dendrites [31,45]. The current data show that similar changes were also present in the hippocampus. Previous studies showed that changing the diurnal cycle results in changes in hippocampal neurons. In comparison with a normal light to dark ratio (14 h at~150 lux and 10 h at 0 lux), Nile grass diurnal rats kept in dim light at night (14 h at~150 lux and 10 h at 5 lux) for 21 days exhibited reduced dendritic length in CA1 and dentate gyrus hippocampus neurons. These animals also showed signs of depressive behavior during the dark phase [46]. Similarly, hamsters exposed to dim light throughout the night showed reduced dendritic length in CA1 neurons coupled with depressive behaviors. This effect was worse with blue and white light compared with red light [47,48]. Finally, compared with a normal light–dark cycle, constant light exposure led to altered hippocampal neurogenesis, which was associated with impaired spatial learning and memory in the Morris water maze task in rodents [49].

Ours and other studies have shown that the retina is severely damaged by prolonged exposure to short-wavelength light [14,31,50]. Even after the degeneration of photoreceptors, there was evidence of ongoing apoptotic activity, as indicated by caspase 3 staining of the inner retinal and retinal ganglion cells [31]. It is likely that such injury would result in altered input into the hippocampus from ipRGCs. The current data showed that BBLs provided some protection against high-level blue-light exposure. This was particularly apparent with BBLs providing better hippocampal cell complexity and fewer unhealthy neurons compared with the LE group. Both lenses employed in this study appeared to have similar effects. 

A number of limitations are noteworthy. This study employs relatively high levels of blue-light exposure which, over a short period, provide a proof-of-principle that non-visual targets in the brain are also impacted by retinal changes induced by short-wavelength light exposure. Studies employing more moderate light levels for longer periods of time would perhaps more closely model what would be encountered by humans exposed to digital devices as well as household/office lighting. The current study has not directly examined retinal ipRGCs, and as such is unclear how changes to retinal input into the hippocampus might impact spatial learning and memory. Here, we employed a behavioral test that has a visual component, which could have confounded the results. Thus, the behavioral outcomes need to be interpreted with caution. Future studies employing this approach would benefit from the inclusion of false-positive control trials. Future investigations could also examine alterations to glutamatergic synapses of hippocampal neurons, which are known to be closely related to behavioral outcomes. 

## 5. Conclusions

Constant and cumulative exposure to high levels of short-wavelength light generated by LEDs resulted in alterations in rat hippocampal neurons (CA1 and CA3), as revealed by morphometric and histopathological assessments. Behavioral analysis revealed impaired spatial learning and memory. This impact could be ameliorated using commercially available BBLs, particularly DB lenses, due to their filtering properties. Further studies are needed to understand the impact of chronic exposure to mild levels of short-wavelength light commonly encountered on visual and non-visual targets of retinal ganglion cells in the rat brain. 

## Data Availability

The data presented in this study are available on request from the corresponding author.

## Supplementary Materials

The following supporting information can be downloaded at: https://www.mdpi.com/article/10.3390/ijerph191912922/s1, Figure S1. (A). Representative photomicrograph of labeled hippocampus regions. (B). Healthy neurons show a pale cytoplasm (C). Irregularly shaped hyper dense neuronal cell bodies (pyknotic cell) indicate a degenerated neuron.
