# Supplementary material for: Blue-Light-Blocking Lenses Ameliorate Structural Alterations in the Rodent Hippocampus
Source: Int J Environ Res Public Health. 2022 Oct 9;19(19):12922. doi: 10.3390/ijerph191912922 (PMC9564388; doi:10.3390/ijerph191912922)
Supplement: Supplementary file 1 [file ijerph-19-12922-s001.zip › ijerph-1912828-SI.pdf]

Supplementary Figure S1

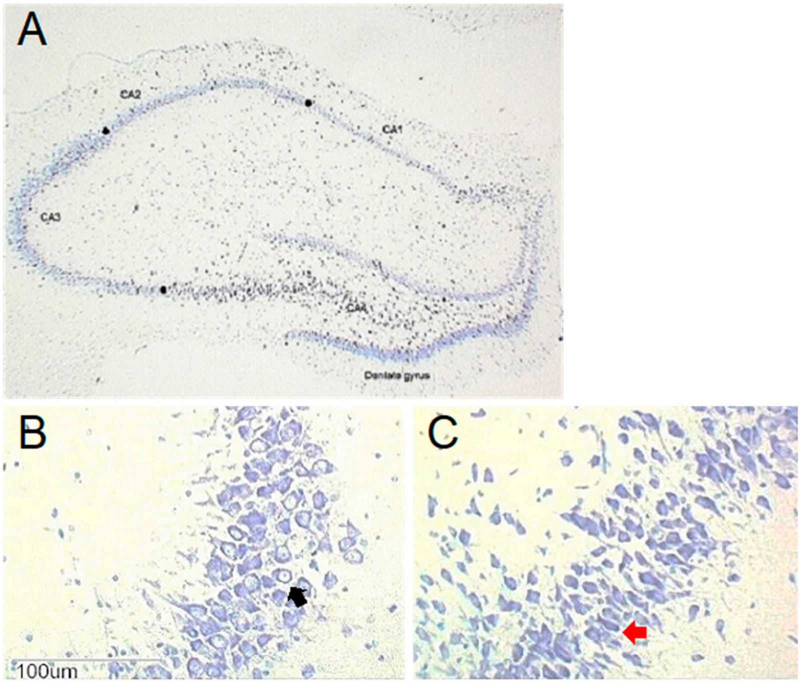

(A). Representative photomicrograph of labeled hippocampus regions. (B). Healthy neurons show a pale cytoplasm (indicated in black arrow) (C). Irregularly shaped hyper dense neuronal cell bodies (pyknotic cell) indicate a degenerated neuron (indicated in red arrow).
